# Small Protein Enrichment Improves Proteomics Detection of sORF Encoded Polypeptides

**DOI:** 10.3389/fgene.2021.713400

**Published:** 2021-10-15

**Authors:** Igor Fijalkowski, Marlies K. R. Peeters, Petra Van Damme

**Affiliations:** ^1^ iRIP Unit, Laboratory of Microbiology, Department of Biochemistry and Microbiology, Ghent University, Gent, Belgium; ^2^ BioBix, Department of Data Analysis and Mathematical Modelling, Ghent University, Gent, Belgium

**Keywords:** SEPs, sORFs, proteomics, peptidomics, riboproteogenomics, amphipathic polymers

## Abstract

With the rapid growth in the number of sequenced genomes, genome annotation efforts became almost exclusively reliant on automated pipelines. Despite their unquestionable utility, these methods have been shown to underestimate the true complexity of the studied genomes, with small open reading frames (sORFs; ORFs typically considered shorter than 300 nucleotides) and, in consequence, their protein products (sORF encoded polypeptides or SEPs) being the primary example of a poorly annotated and highly underexplored class of genomic elements. With the advent of advanced translatomics such as ribosome profiling, reannotation efforts have progressed a great deal in providing translation evidence for numerous, previously unannotated sORFs. However, proteomics validation of these riboproteogenomics discoveries remains challenging due to their short length and often highly variable physiochemical properties. In this work we evaluate and compare tailored, yet easily adaptable, protein extraction methodologies for their efficacy in the extraction and concomitantly proteomics detection of SEPs expressed in the prokaryotic model pathogen *Salmonella typhimurium* (*S. typhimurium*). Further, an optimized protocol for the enrichment and efficient detection of SEPs making use of the of amphipathic polymer amphipol A8-35 and relying on differential peptide vs. protein solubility was developed and compared with global extraction methods making use of chaotropic agents. Given the versatile biological functions SEPs have been shown to exert, this work provides an accessible protocol for proteomics exploration of this fascinating class of small proteins.

## 1 Introduction

Despite their proven involvement in a plethora of cellular processes, small open reading frame (sORF) encoded polypeptides (SEPs) have been historically understudied. In prokaryotes, these small proteins with arbitrarily lengths of up to 100 amino acids (aa) have been shown, among others, to be involved in cellular metabolism, antibiotic resistance, infection and to make up part of the stress response machinery (Rowland et al., 2004; Wadler and Vanderpool, 2007; Hemm et al., 2010; Hobbs et al., 2012). Interestingly, in *Mycoplasma pneumoniae*, SEPs have been shown to be the most frequent essential genomic elements (Lluch-Senar et al., 2015). Given the exponential growth in the number of sequenced genomes, automated genome annotation pipelines have superseded manual curation efforts (Warren et al., 2010). Despite their great utility, due to arbitrary length cut-offs aimed at limiting overprediction and the intrinsic difficulty of sequence analysis of sORFs, the full diversity of many, even well studied, bacterial genomes remains incomplete and underappreciated by automated annotation tools (Dinger et al., 2008; Samayoa et al., 2011; Baek et al., 2017).

Rapid advances in the domain of genomics, particularly through continuous development of ribosome profiling (Ribo-seq), a technology based on massive-parallel sequencing of mRNA fragments encapsulated within actively translating ribosomes, facilitated significant progress towards the discovery of the full repertoire of translated bacterial open reading frames (ORFs) (Ingolia et al., 2009; Giess et al., 2017; Ndah et al., 2017). Recently, a variant of this technique has been reported, allowing for selective immobilization and profiling of initiating bacterial ribosomes, providing yet another high-resolution tool for precise delineation of translated genomic regions (Meydan et al., 2019). These approaches have additionally provided ample evidence of the ubiquitous nature of sORF translation in bacterial genomes, yet proteomic detection of their protein products and their functional validation remain a significant challenge (Ndah et al., 2017; Miravet-Verde et al., 2019; Bartel et al., 2020).

Many presumed justifications of the poor detectability of SEPs using mass spectrometry (MS) have been proposed over the years. With classical bottom-up proteomics approaches relying on the presence of SEP sequences in the search database used to interrogate MS-data, the current state of bacterial genome annotations prevents their comprehensive detection when standard and universal databases are being used. However, putative sequences of novel proteins detected with the aid of ribosome profiling can be included in search databases used to interrogate proteomics datasets. Such efforts are collectively referred to as riboproteogenomics (Ndah et al., 2017; Fijalkowska et al., 2020). Importantly, intrinsically to their short length, SEPs can only produce a very limited number of unique tryptic peptides—2,5% of all theoretically possible tryptic peptides—despite constituting 10% of all annotated proteins, clearly hindering their identification (Carr et al., 2004; Bartel et al., 2020). Moreover, more than half of the putative *E. coli* sORFs have been predicted to encode transmembrane SEPs with a considerable number of SEPs predicted to be located in the inner membrane (Fontaine et al., 2011). In line, in various other bacterial species, including pathogenic bacteria, SEPs are on average more hydrophobic as compared to the rest of the proteome (Garai and Blanc-Potard, 2020). The bacterial transmembrane domain containing SEPs currently characterized have frequently been found to interact directly with larger protein complexes, thereby regulating cellular functions or alternatively, by functioning as membrane associated toxin-antitoxin molecules (Fozo et al., 2008). These specific biochemical properties of SEPs additionally hinder comprehensive SEP extraction and MS-detectability when making use of commonly employed proteomics protocols (Ibrahim et al., 2007; Hemm et al., 2008). The potential membrane localization of a large subset of SEPs might further suggest that employment of specific, MS-compatible, membrane protein enrichment protocols could facilitate their improved proteomics detection (Popot et al., 2011).

Albeit these properties of sORFs and their resulting SEPs deserve further investigation in order to fully elucidate their impact on the identification of small proteins, many efforts have been undertaken to optimize proteomics strategies employed to enrich for small proteins (Hu et al., 2007; Klein et al., 2007; Saghatelian and Couso, 2015; Bartel et al., 2020). Physical segregation of small and large proteins has been attempted by a plethora of different approaches. Gel or membrane filtration steps, Gel-Eluted Liquid FRaction Entrapment (GELFREE) fractionation systems and reversed phase C8 solid phase extraction (SPE) cartridges have all been used for MW-based protein fractionation, and thus separation of SEPs from larger proteins (Hu et al., 2007; Klein et al., 2007). Recently, a novel approach relying on acetonitrile-based precipitation to deplete proteins with masses above ∼15 kDa has been employed, overall resulting in more confident identification of several small proteins based on increased spectral quality (with fewer interfering, non-assigned peaks) and increased number of peptide to spectrum matches (PSMs), but generally, without new identifications (Cassidy et al., 2019). A comprehensive comparison of 14 available small protein enrichment protocols used to analyze a simplified human gut microbiota model system could confirm the utility of C8 cartridges and GELFREE fractionation as effective approaches by identifying 79 yet uncharacterized small proteins (including SEPs) without prior proteomic evidence (Petruschke et al., 2020).

Further, the use of alternative digestion protocols has been proposed to further facilitate the discovery of SEPs and to increase the number of peptides produced (Kaulich et al., 2021). Creating an *in silico S. typhimurium* tryptic digestion map for all annotated (genome assembly ASM21085v2; 2 missed cleavages allowed) proteins results in 4420 identifiable SEP-derived peptides falling within typical MS-detection limits (mass range of 600–4,600 Da, ≥7 aa), originating from 423 out of 476 annotated *S.* Typhimurium SEPs. This, in contrast to the over 220,000 possible tryptic peptides originating from all annotated proteins, which lowers the detection probability of SEPs. In theory, this number can be improved by utilization of chymotrypsin yielding ∼6,200 theoretically identifiable chymotryptic peptides covering 458 SEPs. Despite this fact, trypsin is still strongly positioned as a go-to protease in proteomics research due to its high specificity, consistent cleavage frequency and high compatibility with MS-detection mainly due to the high efficiency of ionization of the C-terminally charged peptides produced during electrospray ionization (ESI) preceding MS-detection (Giansanti et al., 2016).

In case of eukaryotic genomes, customized, ribosome profiling (protein synthesis-based) databases have been created to host the translated repertoire of putative (s)ORFs for mass spectrometry-based identification, yet corresponding solutions are less mature for bacterial genomes (Crappé et al., 2015; Olexiouk et al., 2016; Brunet et al., 2019). Recently, a first large-scale machine learning-aided sequence analysis effort of prokaryotic genomes provided 109 putative small ORFome predictions across the bacterial phylogeny (Miravet-Verde et al., 2019). We and others have also shown that the use of non-redundant tryptic peptide databases based on bacterial genome sequences translated in all six frames, in conjunction with state-of-the-art high resolution MS only increases the search space modestly (∼4-fold increase in case of *S. typhimurium* in contrast to the over 400-fold inflation for the human genome (Zhu et al., 2018) and can thus conveniently be implemented, also for the reliable proteogenomic identification of SEPs, when combined with modern, robust post-processing tools like Percolator (Käll et al., 2007; Omasits et al., 2017; Willems et al., 2020).

In this work, we present an optimized method for proteomic detection of SEPs in the well-studied model bacterial pathogen *S. typhimurium*. More specifically, we compare commonly employed total protein extraction protocols based on chaotropic agents-urea and guanidine hydrochloride (GuHCl) and propose a complementary use of the amphipathic polymer amphipol A8-35 to deplete larger proteins, while concomitantly enriching for small proteins in the supernatant, thereby improving detection of SEPs.

## 2 Materials and Methods

### 2.1 Bacterial Culture

The *S. enterica* serovar Typhimurium (*S. typhimurium*) wild-type strain SL1344 (Genotype: hisG46, Phenotype: His(-); biotype 26i) was obtained from the *Salmonella* Genetic Stock Center (SGSC, Calgary, Canada; cat n° 438). Bacterial growth was performed using liquid Miller formulation of LB medium (10 g/L Bacto tryptone, 5 g/L Bacto yeast extract, 10 g/L NaCl). In quadruplicates, single colonies were picked from LB/agar plates and propagated in 2 ml of LB medium overnight (37°C, 180 rpm). Precultures were used to inoculate the bacterial cultures at a 1:100 dilution, and cultures grown until an optical density (OD_600_) of 0.8 was reached (representing the late phase of exponential growth). Aliquots of 10 ml culture were centrifuged for 15 min at 5,000 g and 4°C, washed with 25 ml of phosphate-buffered saline (PBS) and centrifuged again for 15 min at 5,000 g and 4 °C. After removing the PBS, the pellets were frozen in liquid nitrogen and stored at −80°C until further use.

### 2.2 Protein Extraction and MS-Analysis

**FIGURE 1:**
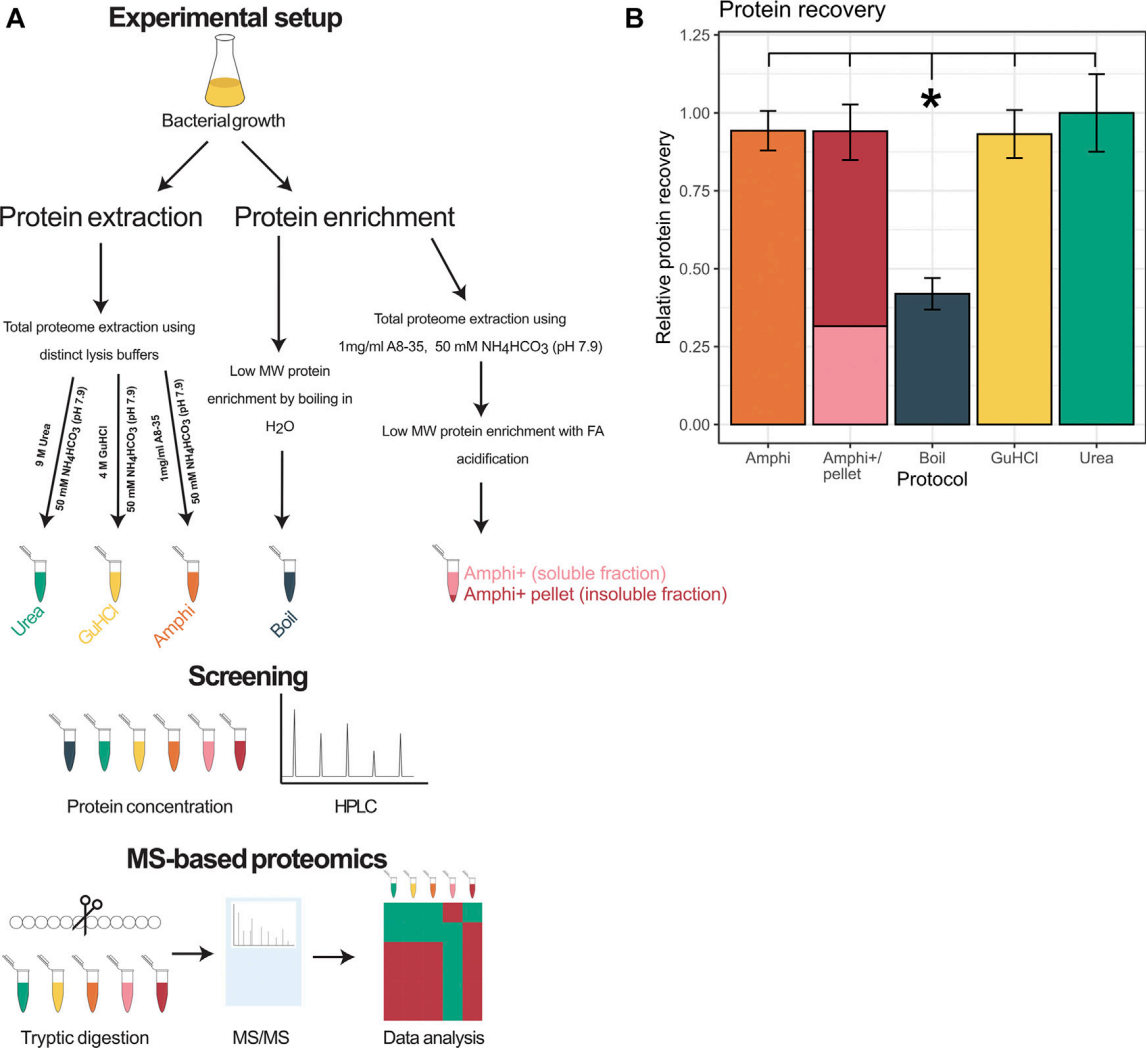
Overview of the experimental setup and protein extraction efficiency. **(A)** Workflow performed in this study. **(B)** Protein recovery using extraction methods investigated in this study shown relative to the extraction method with highest yield of protein recovered (urea).

In total 3 total protein extraction setups have been investigated (experimental design presented in Figure 1A), namely urea facilitated protein extraction [9 M urea in 50 mM ammonium bicarbonate (NH_4_HCO_3_) (pH 7.9); urea], guanidine hydrochloride facilitated protein extraction [4 M GuHCl in 50 mM NH_4_HCO_3_ (pH 7.9); GuHCl) and amphipol A8-35 aided protein extraction (1 mg/ml amphipol in 50 mM NH_4_HCO_3_ (pH 7.9); Amphi]. Further, an optimized complementary enrichment strategy utilizing acidification of amphipol A8-35 total protein extracts has been performed yielding two additional sample types; acidified amphipol supernatant samples enriched in low molecular weight (MW) proteins (Amphi+) and an acidified amphipol insoluble, pelleted fraction enriched in high MW protein (Amphi+ pellet). By acidification of Amphi samples, protein solubility decreases and high MW proteins co-precipitate with the protonated amphipols while stabilized short proteins remain in solution (Ning et al., 2014). More specifically, Amphi samples were acidified with 5% formic acid (FA) till pH 3.0, centrifuged for 10 min at 16,000 g and 4°C, and both supernatant (Amphi+) and pellet (Amphi+ pellet) recovered for downstream analysis. Finally, a commonly used procedure based on boiling of protein extracts in water has been employed to serve as a reference sample to more globally assess protein extraction efficiencies (Ma et al., 2014; Ma et al., 2016). All protocols have been applied in quadruplicates.

Bacterial pellets were resuspended in 600 µl of respective resuspension buffers. In case of amphipol, the samples were vortexed and incubated for 10 min at room temperature (RT) before further processing. In case of boiling, cell pellets were resuspended in preheated (95°C) water and boiled for 10 min before further processing. Mechanical disruption was performed by 3 repetitive freeze-thaw cycles using liquid nitrogen followed by 2 min sonication on ice (Branson probe sonifier output level 4 with 40% duty cycle; 3 × 30 s; 1 s pulses). Lysates were cleared by centrifugation at 16,000 g for 10 min at 4°C and the supernatant was transferred to a clean Eppendorf.

Protein concentrations of soluble samples were measured using BCA (Pierce). 400 µg of protein was brought to 1 mg/ml with respective lysis buffers and the samples precipitated overnight with 4× volumes (1.6 ml) of −20°C acetone. The precipitated protein was collected by centrifuging for 15 min at 3,500 g (4°C), pellets were washed twice with −20°C 80% acetone and air-dried upside down for ∼10 min at room temperature until no acetone odor remained. The dried pellets (alongside Amphi+ pellets) were resuspended in 200 µl TFE (2,2,2-trifluoroethanol) digestion buffer (10% TFE, 100 mM NH_4_HCO_3_) and sonication applied using a Branson probe sonifier (output 10–15, 0.5 s pulses) until a homogenous suspension was formed. All samples were digested overnight at 37°C using MS-grade trypsin (Promega, Madison, WI, United States) (enzyme/substrate of 1:100 w/w) while mixing (550 rpm). The tryptic digest of amphipol pellets were acidified with 5% FA to reach pH 3.0 for amphipol removal, and all other samples acidified with TFA to a final concentration of 0.5%. Samples were cleared from insoluble particulates by centrifugation for 10 min at 16,000 g (4°C) and 100 µl of the supernatant was transferred to clean tubes. Subsequently, uniform methionine oxidation to methionine sulfoxide was performed by the addition of H_2_O_2_ to a final concentration of 0.5% for 30 min at 30°C. Subsequently, pipette tip solid phase extraction (SPE) of peptides was performed (Bond Elut OMIX 100 µl C18 tips (Agilent)) according to the manufacturer’s instructions. In brief, the pipette tips were conditioned by aspirating the maximum pipette tip volume (100 µl) of H_2_O:ACN (50:50, v/v) and the solvent discarded. After equilibration with 3 maximum pipette tip volume of 0.1% TFA in HPLC-grade water, 100 µl of the acidified samples were dispensed and aspirated for 10 cycles for maximum binding efficiency. The tips were washed 3 times with the maximum pipette volume of 0.1% TFA in H_2_O:ACN (98:2, v/v) and the bound peptides eluted in LC-MS/MS vials with the maximum pipette tip volume of 0.1% TFA in H_2_O:ACN (30:70, v/v) by aspirating and dispensing the buffer twice. The peptide samples were vacuum-dried in a SpeedVac concentrator and dissolved in 20 µl of 2 mM tris-(2-carboxyethyl)-phosphine (TCEP) in H_2_O:ACN (98:2, v/v). Samples were subjected to LC-MS/MS analysis using an UltiMate 3000 RSLC nano HPLC (Dionex, Amsterdam, Netherlands) in-line connected to a Q-Exactive HF mass spectrometer (Thermo Fisher Scientific Inc., Bremen, Germany). The mass spectrometer was operated in data-dependent, positive ionization mode as described before (Gawron et al., 2016).

### 2.3 Data Analysis

The obtained MS-data was searched using MaxQuant software (version 1.6.10.43) using the ASM21085v2 SL1344 *S. typhimurium* genome annotation and assembly. The methionine oxidation was set as fixed modification and decoy database of reversed protein sequences was used to estimate FDR, and an 1% FDR threshold applied. Minimum peptide length of 7 and a mass window between 600 and 4,600 Da was considered. Trypsin/P was selected as the digestion enzyme. The match between runs function was enabled and proteins were quantified by both the MaxLFQ algorithm and iBAQ algorithm integrated in the MaxQuant software (Tyanova et al., 2016a). Here, a minimum of two ratio counts and only unique peptides were considered for protein quantification. The data was further processed with the use of Perseus software suite (version 1.6.10.50; Tyanova et al., 2016b) and custom R scripts. Hierarchical clustering analysis was performed using Euclidean distance calculation and subsequent GO enrichment analysis was performed using Fisher exact test (at FDR 0.02) on relative GO term occurrence in the function of distinct protein clusters. GO terms were obtained from UniProt (proteomeID UP000008962). Publicly available ribosome profiling datasets were downloaded from NCBI’s Gene Expression Omnibus (accession number GSE91066) (Ndah et al., 2017).

### 2.4 RP-HPLC Chromatographic Peptide Profile Inspection of Hydrophobicity Bias

In order to test for possible biases in peptide solubility of the employed protein extraction methods, 4 nmol of a dried equimolar peptide mixture (cfr. pepmix) was resuspended in 800 µl of the above described extraction buffers to prepare samples for reverse phase (C18) HPLC analysis. The pepmix was composed out of 5 peptides with different RP-HPLC elution profiles, thus presenting a range of hydrophobicities (Table 1). Amphi, urea and GuHCl, Amphi+ (soluble fraction after FA acidification) and boiled samples were processed according to above described proteomics protocols (Figure 1A) and acidified with TFA to reach a final concentration of 0.5% or, in case of Amphi+ samples, acidified with FA to reach a final pH of 3.0. The acidification step was followed by a centrifugation for 10 min at 16,000 g and transfer of the supernatant in HPLC vials. 100 µl was injected corresponding to the equivalent of ∼0.5 nmol of input peptide material. A linear gradient making use of solvent A (2% ACN, 0.01% TFA, pH 3) and solvent B (70% ACN, 0.01% TFA) was applied for peptide separation over 100 min. The sample separation was performed using a C18 column, internal diameter (I.D.) of 2.1 mm, running at an on-column micro flow-rate of 80 μl/min using an HPLC setup as described before (Staes et al., 2008).

**TABLE 1:** Synthetic peptides used Reverse-Phase High-Performance Liquid Chromatography (RP-HPLC) investigation of peptide solubilization biases in selected protocols under study. Physiochemical properties of synthetic peptides used for RP-HPLC investigation of potential biases present in the evaluated protein extraction protocols.

Peptide	Sequence	MW (Da)	Hydrophobicity (GRAVY)	Length	Aliphatic index	Charge	pI
1	NH2.GYHLNEEGTR.OH	1,174.54	−1.72	10	39	2.07	5.49
2	NH2.IILEDYHDHGLLR.OH	1,592.83	−0.21	13	150	2.76	5.36
3	NH2.LLSSSNELVTR.OH	1,217.66	0.09	11	132.73	1.13	6.41
4	NH2.VAGLLEDTFPGLLGLR.OH	1,669.94	0.8	16	0.8	0.94	4.18
5	NH2.IAPAVVHIELFR.OH	1,363.8	1.23	12	162.5	2.13	7.55

## 3 Results

In order to comprehend and potentially aid the MS-based small proteome coverage of *S. typhimurium*, we compared three widely employed global protein extraction methods-making use of the chaotropic agents urea (urea), guanidine hydrochloride (GuHCl) and an extraction making use of amphipol (Amphi), a mild surfactant with amphipathic characteristics. Moreover, we investigated the commonly used small protein enrichment strategy of boiling (Boil) known to favor extraction of small proteins, besides a newly designed setup potentially enriching for small MW proteins (i.e., an extraction making use of amphipol, followed by an FA precipitation step to deplete for high MW protein) (Figure 1A). In case of the amphipol enrichment strategy based on acid precipitation, both the supernatant (Amphi +; the soluble fraction) and resulting pellet after precipitation (Amphi+ pellet; the insoluble fraction) were considered for further analysis. The protocols tested resulted in good and comparable (total) protein recovery with a notable exception of the boiled samples with less than half (42%) of the maximum amount of total protein recovered (Figure 1B).

**FIGURE 2:**
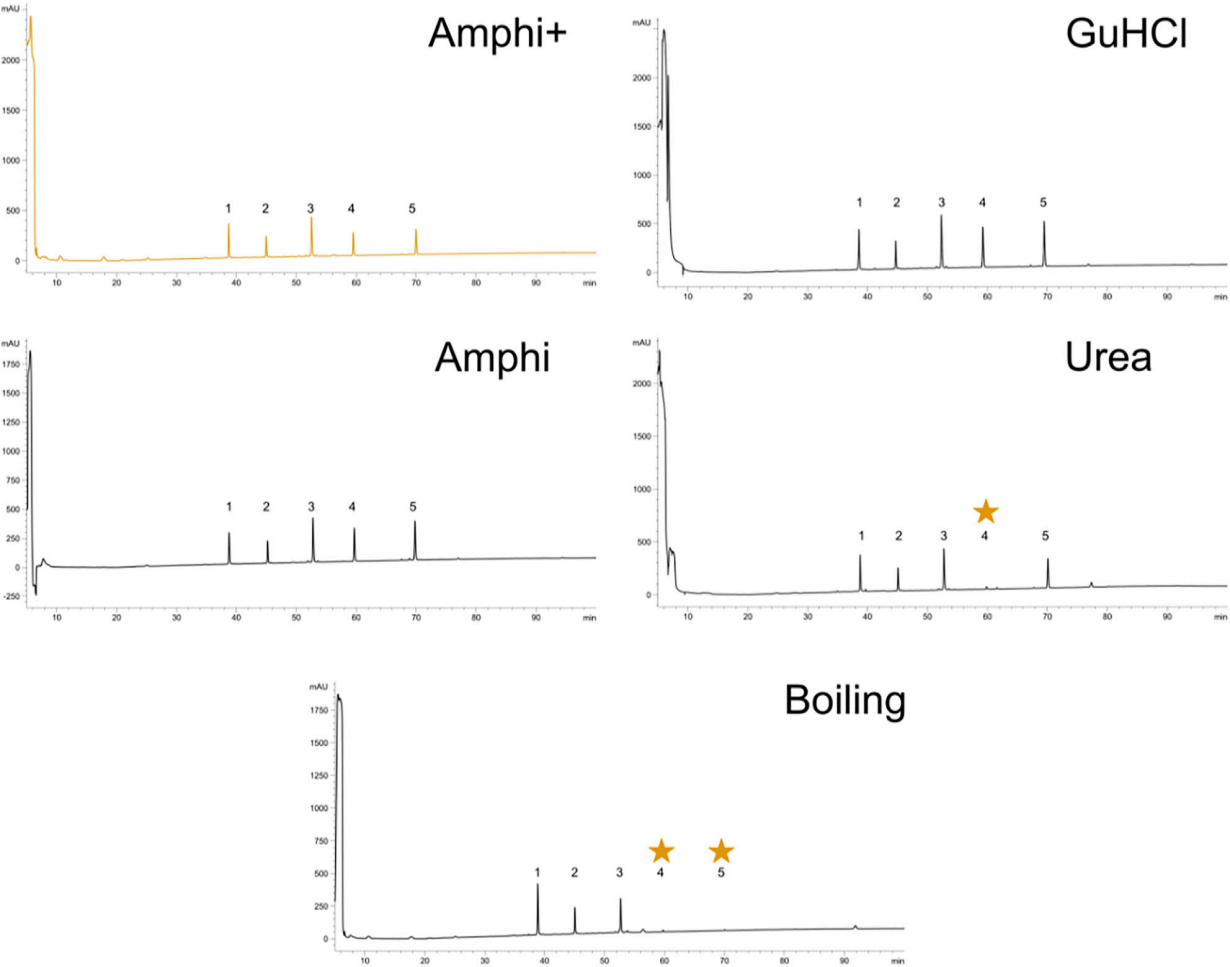
Reverse-Phase High-Performance Liquid Chromatography (RP-HPLC) investigation of peptide solubilization biases in selected protocols under study. RP-HPLC chromatograms of a synthethic peptide mix processed with the 5 experimental procedures described in this study (graph headers). Arbitrary units of absorption at 280 nm are presented across the 100 min ascending ACN gradient applied. The expected elution interval of missing peptides are indicated with an asteriks. A representative replicate is shown for each setup.

To further investigate potential intrinsic biases for SEP detection in the extraction protocols employed, we tested the protocols to assess the recovery of a panel of synthetic peptides indicative of short SEPs with varying hydrophobicity indexes (Table 1). To do this, we performed reverse phase chromatography (RP-HPLC) of the synthetic peptide mixture processed with each protocol under study in triplicate. As evident from the representative chromatograms obtained (Figure 2), all experimental procedures under study recovered the investigated peptides to a comparable degree with a notable exception of the urea and the boiling protocol. In case of boiling, the two most hydrophobic peptides were lost while for the urea protocol, only the fourth eluting peptide (NH2.VAGLLEDTFPGLLGLR.OH, marked with a star in Figure 2)—characterized by a hydrophobicity of 0,8 on GRAVY scale and eluting around 60 min—was not recovered. In contrast to the boiling method, this observation seems not to be related to peptide hydrophobicity exclusively, as the more hydrophobic peptide eluting at a later time (peptide 5) could be observed. This observation indicates potential intrinsic differences between chaotropic agents in the efficiency of peptide extraction/recovery. In fact, since the differentially extracted peptide displays the lowest isoelectric point from all 5 peptides present in the mixture (4.18), its absence in the extract can likely be attributed to differences in ionic characteristics between urea and GuHCl (Monera et al., 1994). With GuHCl releasing ions masking both positively and negatively charged amino acid side chains, reduction in electrostatic interactions can be expected to potentially aid the dissolution and thus recovery of the peptide (Shaw et al., 2001).

**FIGURE 3:**
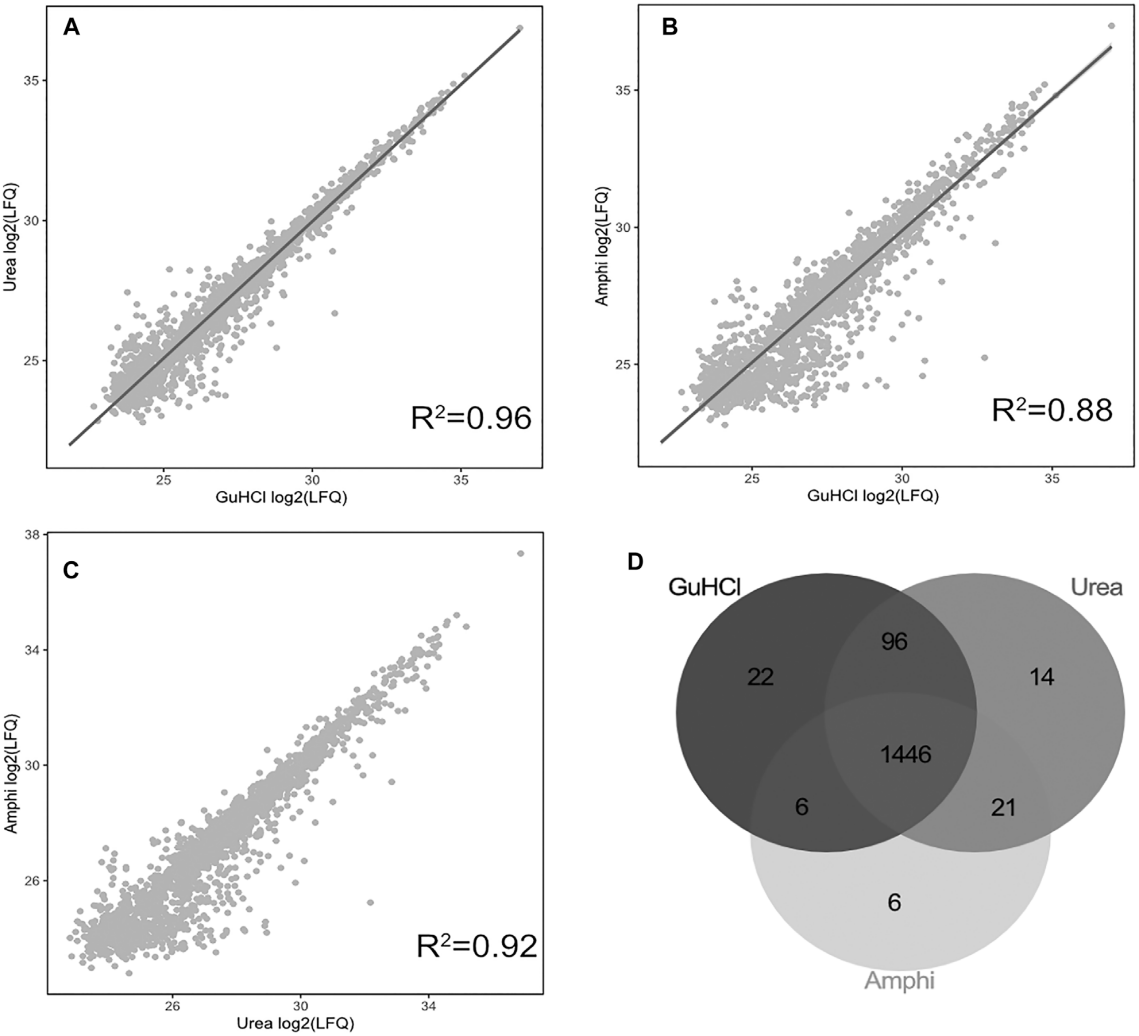
Proteomics results using 3 global protein extraction methods. **(A–C)** Correlation plots of log2 LFQ intensities across 3 different experimental setups for global protein extraction (amphi, urea and GuHCl) studied by shotgun proteomics. **(D)** Venn diagram illustrating the overlap in proteins identified and quantified across the 3 extraction methods (in grey scale).

**FIGURE 4:**
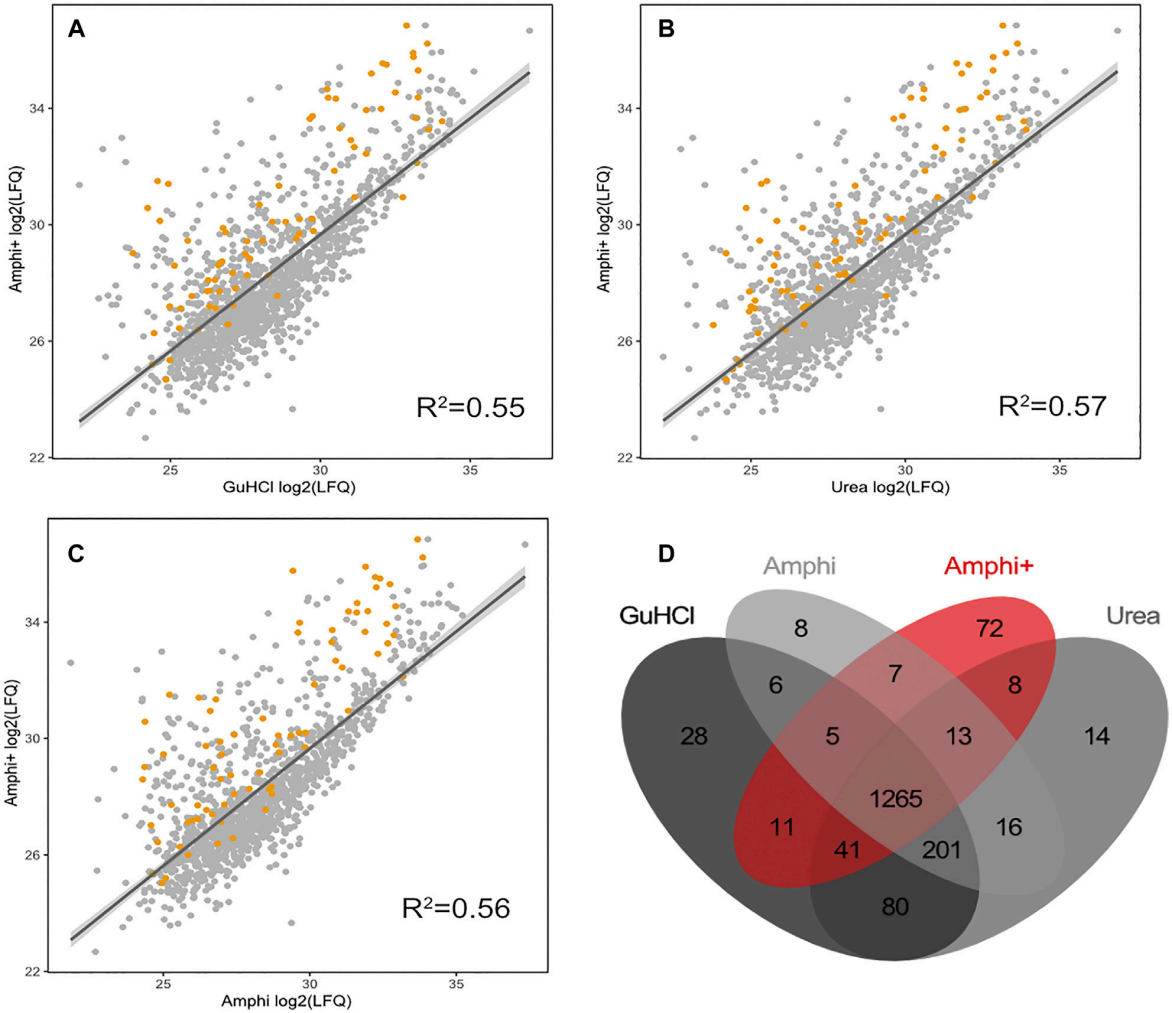
Proteomics results of Amphi+ samples in comparison to other investigated extraction methods. **(A–C)** Correlation plots between log2 LFQ intensities of amphipol enriched samples (Amphi+) and 3 total protein extraction methods tested (amphi, urea and GuHCl). Identified annotated SEPs are highlighted in orange. **(D)** Venn diagram illustrating overlap in proteins quantified across all 3 total extraction methods (in grey scale) and compared to the Amphi + setup (red).

**FIGURE 5:**
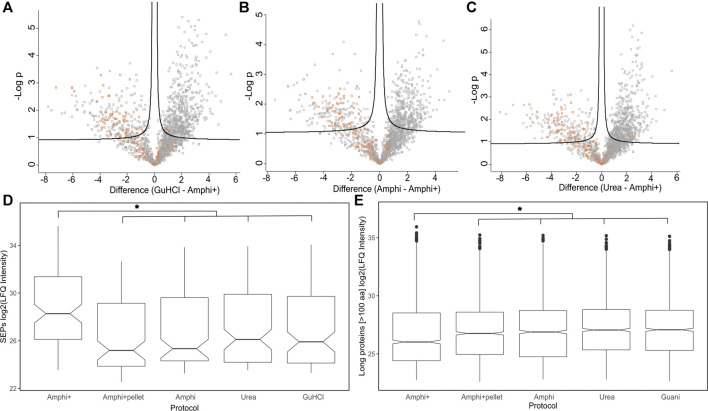
Differential analysis of proteins enriched in Amphi+ setup. **(A–C)** Volcano plots illustrating differential protein abundances between investigated extraction protocols. The 3 total extraction methods (amphi, urea and GuHCl) are pair-wise compared to enriched amphipol samples (Amphi+) and the relation between log of *p*-value is plotted against fold change in protein abundance. Solid curved lines represent a significance threshold of 1% FDR (S0 = 0.1). SEPs are highlighted in orange. **(D)** Boxplot illustrating log2 LFQ intensities of SEPs identified in samples across all investigated experimental setups. Significant difference is marked with asterix (*p*-value <0.01, t-test) **(E)** Boxplot illustrating log2 LFQ intensities of proteins longer than 100 aa identified in all investigated experimental setups. Significant difference is marked with asterix (*p*-value <0.01, t-test).

**FIGURE 6:**
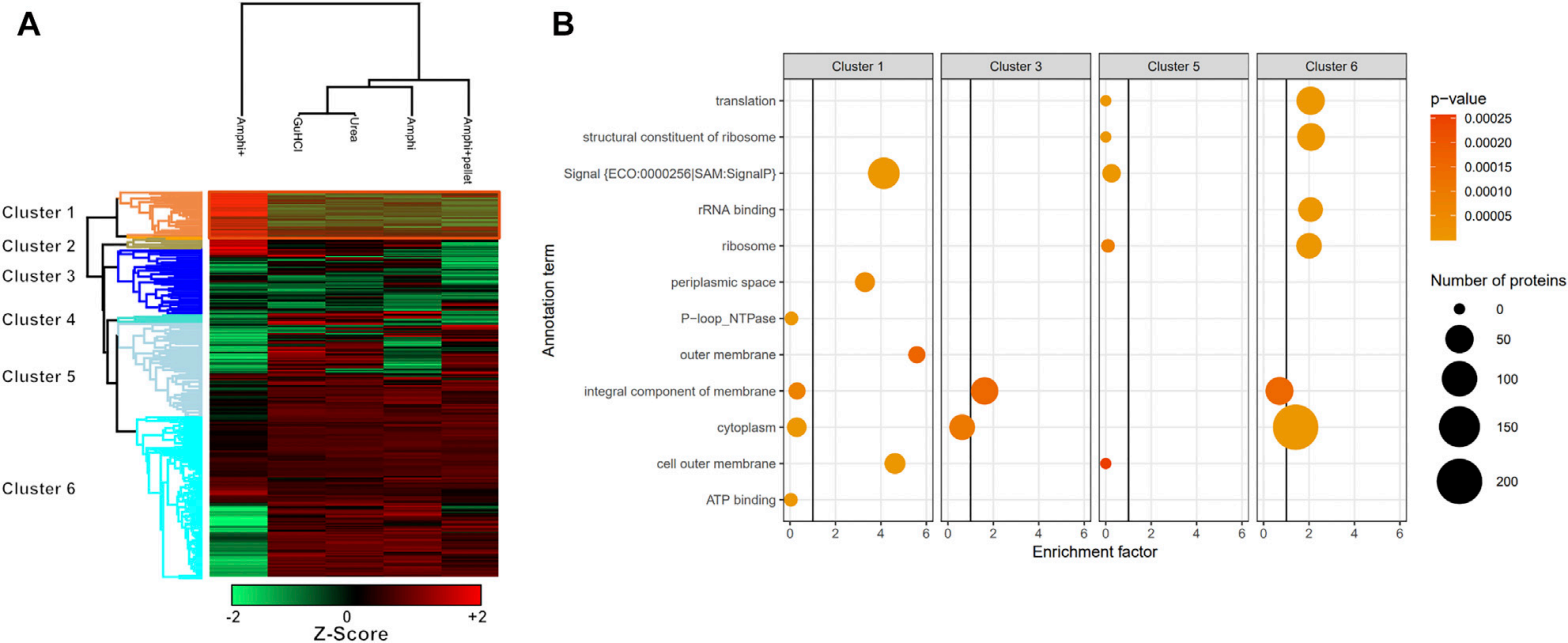
Hierarchical clustering and GO enrichment analysis. **(A)** Hierarchical clustering of investigated experimental setups with the cluster of proteins significantly enriched in enriched Amphipol (Amphi+) samples (*p*-value <0.01) highlighted in orange (Cluster 1; Supplementary Table S2). Red represents **higher** abundance, green represents **lower** abundance. **(B)** GO analysis results presenting significantly regulated GO terms found in clusters corresponding to panel A. Size corresponds to the number of regulated proteins with a given GO term and color represents the *p*-value scale.

Viewing the significantly lower—and thus very likely highly biased—protein extraction yield and failure to extract hydrophobic peptides the boiling setup was not considered for further comprehensive shotgun LC-MS/MS analysis. Subjecting the other protein extraction protocols to proteomics analysis (urea, GuHCl, Amphi, Amphi+-soluble fraction after FA acidification, Amphi+ pellet-the insoluble fraction after FA acidification, all performed in quadruplicates) and considering only unique (non-redundant) peptide identifications, resulted in the identification of 2171*S.* Typhimurium proteins, corresponding to 46% of the annotated *S.* Typhimurium proteome (Supplementary Table S1A). For relative protein quantification and to reveal (potential) differences in protein/SEP extraction efficiencies across the different setups, quality filtering for proteins with valid values in at least 3 replicates of at least one experimental setup was performed, overall leaving 1,676 proteins (35% of the annotated proteome) for downstream analysis (Supplementary Table S1B). Biological replicates among all total protein extraction setups display high cross-correlation indicating high reproducibility and robustness of the used protocols (Supplementary Figure S1). As evident from the correlation plots displayed in Figures 3A–C, the protein abundance (LFQ intensities) data across all three total protein extraction methods correlates well (average *R* ^2^ of 0.93) and only a modest number of unique protein identifications per condition is observed (Figure 3D). GuHCl extraction has provided the most comprehensive proteome view displaying the highest overlap with alternative extraction methods tested, as here 1785 proteins were identified of which 1,570 proteins were reliably quantified (Figure 3D). Besides, 130 unique proteins were identified (2 uniquely quantified proteins) (Supplementary Table S1). This finding is in line with results previously reported in Ndah et al. (2017). Urea based extraction performs similarly, providing identification of 1720 proteins (quantification of 1,577 with 56 unique identifications and 1 unique quantification) (Supplementary Table S1) and correlating highly with GuHCl extraction data (*R* ^2^ of 0.95, Figure 3A). As expected, the amphipol enriched (Amphi+; soluble fraction) samples differed most significantly from all total protein extraction methods as evident from correlation scores (average *R* ^2^ of 0.56) and differential expression analysis (Figures 4A–D, 5A–C, 6A,B). Further, the amphipol enriched pellet samples (Amphi+ pellet; insoluble fraction) display a depletion in proteins found to be upregulated in Amphi+ samples, while otherwise closely resembling Amphi total extraction where no FA acidification was performed (*R* ^2^ of 0.91; Figure 5A).

**FIGURE 7:**
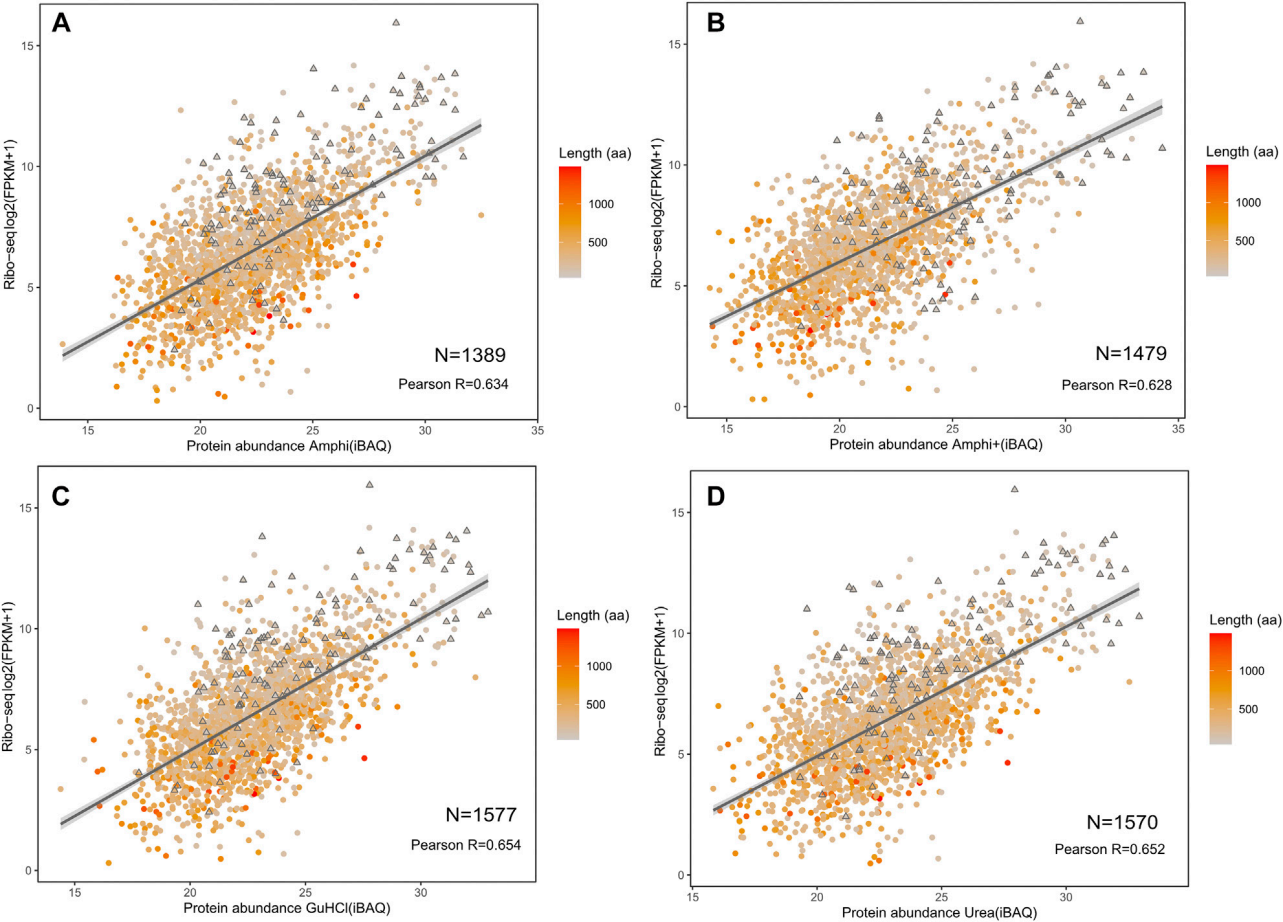
Correlation of investigated protocols with translation data based on ribosome profiling (Ndah et al., 2017). **(A–D)** Correlation plots between log2 fragments per kilobase per milion (FPKMs) translation measurement based on ribosome profiling with LFQ intensities obtained from shotgun proteomics of the amphi, amphi+, urea and GuHCl extraction methods. The color scale represents the length of respective proteins (aa) with SEPs indicated as triangles.

Proteomics results were correlated with publicly available *S. typhimurium* translatomics data (ribosome profiling data obtained in similar growth conditions (OD600 0.6) to investigate potential biases in the proteome representation of the different (total) protein extraction protocols tested (Ndah et al., 2017). Despite overall good iBAQ (intensity Based Absolute Quantification) protein abundance correlations with ribosome profiling (FPKM) ranging from 0.628 till 0.654 (Figure 7), both urea and GuHCl protocols provided a more comprehensive picture of the proteome based on markedly higher number of confidently quantified proteins (1,577 and 1,570 proteins, respectively) as compared to Amphi+ (soluble fraction after FA acidification) and Amphi extraction samples (1,389 and 1,479 proteins, respectively). Interestingly, when observing the relation between translation and protein abundance in function of protein size (color scale Figure 7) we can observe that the Amphi + method only marginally affects the correlation (Figure 7) also for SEPs (highlighted with triangles) (Pearson coef. of 0.628 vs. 0.646 average for other conditions). This suggests that despite effectively enriching for small proteins, the Amphi+ method does however not significantly affect the global quantitative aspect of the proteomic analysis. Noteworthy however, proteins with larger mass generally appear enriched in the proteome over the translatome, samples and vice versa for low mass protein, indicating that iBAQ molecular weight normalization is still biased.

As presented in Figures 4A–C however, the Amphi+ samples displayed increased abundances of SEPs (highlighted in orange) when compared to the global extraction methods tested by its depletion of higher MW proteins. Moreover, the 72 proteins uniquely identified in Amphi + setup included 17 SEPs out of the 140 SEPs identified in total (Figure 4D; Supplementary Table S1). With hierarchical clustering of regulated proteins comparing experimental conditions, a cluster of 215 proteins significantly enriched in Amphi+ samples can be observed (orange cluster in Figure 6A, average protein abundance fold change of 5.98 based on protein quantifications). Interestingly, 57 of these enriched proteins are SEPs, again confirming that Amphi+ protocol enriches for SEPs. Only 19 of these SEPs are currently annotated with a GO term. This clear property of the Amphi+ extraction in facilitating the detection of small proteins is also evident from the average MW plots presented in Figure 5D. Inversely, larger proteins are significantly depleted in Amphi+ samples (Figure 5E). Hierarchical clustering (Figure 6A) further demonstrates the distinct SEP abundance profile in the Amphi+ samples when compared to the total protein extractions investigated. GO enrichment analysis using UniProt GO terms further revealed 12 significantly (Fisher exact test, *p*-value <0.01) differentially regulated annotation terms (Figure 6B; Supplementary Table S2). Cluster 1 comprised proteins upregulated in Amphi+ samples showing a significant enrichment of signal peptide containing proteins (keyword). 71 of such proteins, with an GO enrichment factor of 4.1 were detected. In consequence, signal peptide bearing categories of proteins have been found to be enriched in Amphi+ samples. This included both periplasmic (13 proteins, GO enrichment factor of 3.3) and outer membrane proteins (22 proteins, GO enrichment factor of 4.9). Contrary, cytoplasmic and integral (inner) membrane proteins were depleted in this cluster (12 and 6 proteins respectively, both with an GO enrichment factor of 0.3). Complementary to these findings, clusters 3–6 represent proteins depleted in Amphi+ samples while significantly enriched in all 3 total protein extraction methods tested in this study. To this end, cytoplasmic proteins were significantly enriched in cluster 6 (200 proteins, GO enrichment factor 1.4) while integral (inner) membrane proteins assigned to cluster 3 were detected at higher levels (45 proteins, GO enrichment factor 1.6).

**FIGURE 8:**
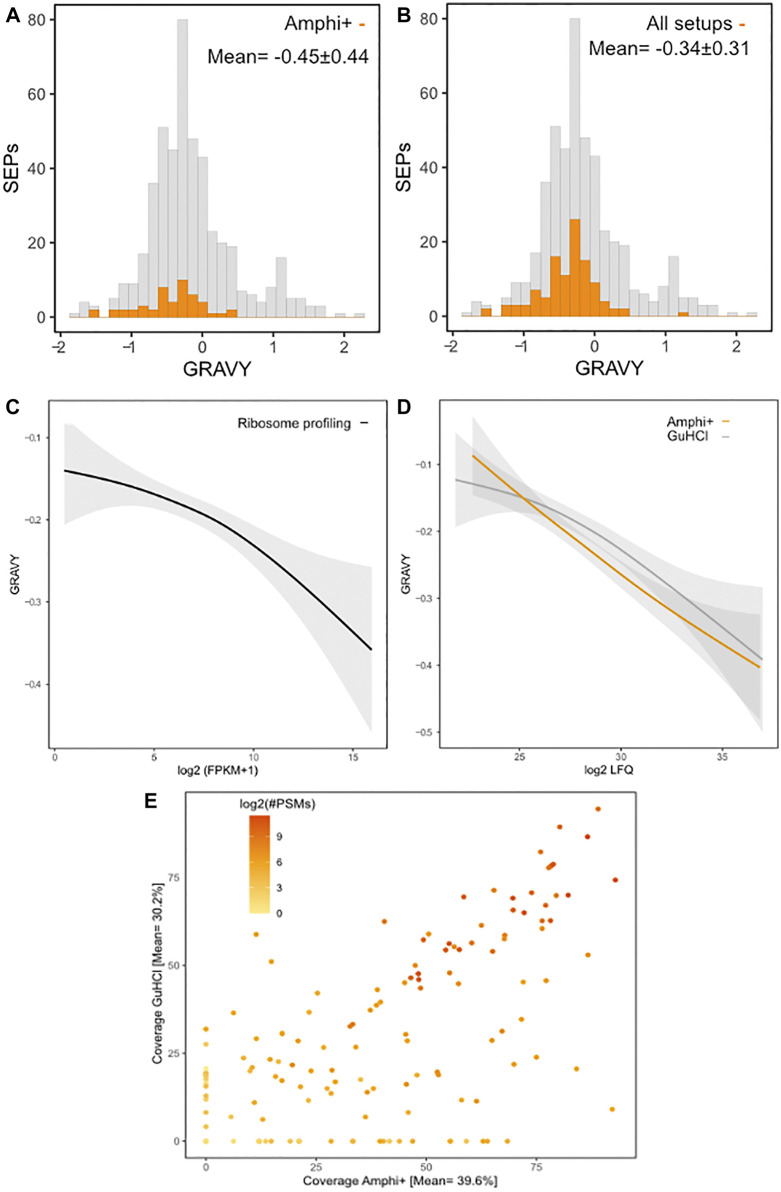
Protein hydrophobicity and proteomic coverage across investigated protocols. **(A)** Histogram of GRAVY hydrophobicity scores of all annotated *S.* Typhimurium SEPs with SEPs with significant (*p*-value < 0.01) higher abundances in Amphi+ samples highlighted in orange. **(B)** Histogram of GRAVY hydrophobicity scores of all annotated *S.* Typhimurium SEPs with all SEPs identified in this study highlighted in orange. **(C)** Regression of the relation between protein hydrophobicity and expression as measured by ribosome profiling (log2 FPKMs). **(D)** Regression of the relation between protein hydrophobicity and expression as measured by proteomics (log2 iBAQ) in Amphi+ (orange) and GuHCl (grey) samples. Colored lines represent the median, greyed out areas represent middle 2 quartiles of data distribution. **(E)** Relation between coverage of SEPs observed in GuHCl vs. Amphi+ setup with a number of PSMs matches represented in log2 scale.

In total 111 out of 498 annotated *S. typhimurium* SEPs were identified across all experimental conditions. SEPs exclusively identified in a specific setup were only recorded in Amphi+ samples (17 unique SEPs) and Amphi total protein extraction samples (2 unique SEPs). Expectedly, the SEPs uniquely identified in Amphi+ were detected with, on average, lower number of peptides (3 vs. 4.7 for all SEPs) and lower number of PSMs (22 vs. 50 for all SEPs) as compared to all SEPs identified, but overall, SEPs were, on average, found enriched in Amphi+ samples (Figures 5A–D). This observation has further been confirmed with a 2-sample t-tests presented in volcano plots in Figure 5 (*p*-value < 0.01). With the demonstrated utility in facilitating SEPs detection, the Amphi + setup however does not seem to clearly enrich for hydrophobic SEPs, despite effectively enriching for outer membrane proteins (Figure 6B), as evident from the distribution of the hydrophobicity scores of SEPs detected using this method compared to all annotated *S.* Typhimurium SEPs (Figure 8A) (Kyte and Doolittle, 1982). Moreover, the hydrophobicity (GRAVY) of SEPs enriched in the Amphi+ setup didn’t differ significantly from the hydrophobicity of SEPs detected across all experimental setups analyzed, and thus could point to a general extraction or MS-based detection bias (Figure 8B). However, when compared to ribosome profiling data, where no hydrophobicity bias is in principle expected, we did observe that GuHCl extraction better reflects the distribution of hydrophobicity scores across the proteome than the amphipol enrichment strategy, especially in the middle section of the hydrophobicity distribution (Figures 8C,D). When comparing Amphi+ in a pairwise fashion to GuHCl, urea and amphipol extraction methods (Figures 5A–C, 6A), respectively 62, 45 and 47 SEPs were found to be significantly enriched in the Amphi+ samples. Interestingly, the Amphi+ setup also provided a markedly increased coverage of SEPs with unique peptides (average 39.6% coverage vs. 30.9% in other protocols; Figure 8E) as well as an increased number of PSMs originating from SEPs (on average 123 versus 50 PSMs in an Amphi+ and GuHCl sample, respectively).

## 4 Discussion

Amphipatic polymers, and amphipol A8-35 in particular, have been used in proteomics efforts for the efficient stabilization of membrane proteins in detergent-free solutions for over a decade now in a broad range of applications (Popot et al., 2011). Amphipol has been described to effectively bind hydrophobic surfaces of transmembrane proteins stabilizing their native structure (Popot, 2010). Moreover, amphipol was previously shown to be useful in precipitation and concentration of protein mixtures in an unbiased manner (Ning et al., 2014). With growing evidence of the ubiquitous nature of short, often transmembrane domain containing SEPs expressed in bacterial cells (i.e., 71 out of 498 annotated *S. typhimurium* SEPs (∼15%) are predicted to contain a transmembrane domain), simple, yet tailored proteomic approaches can readily enhance their proteomic discovery. With systemic efforts aimed at cataloguing such interesting genes, the need for proteomic validation grows stronger than ever as no proteomic evidence could be found so far for a vast majority of novel SEPs identified through genomic efforts, even when making use of integrative in silico and experimental OMICs approaches (Venturini et al., 2020). This fact, puts optimization of custom SEP enrichment protocols at the forefront towards their proteomic validation. In this study we investigated the alternative utility of amphipol; namely the property of enabling the enrichment of SEPs by specifically co-precipitating with larger MW proteins upon acidification, and by performing a comparative analysis with more generally employed total protein extraction methodologies. By depleting larger proteins and enriching SEPs present in proteome extracts we achieve an over four-fold enrichment of SEPs. Considering the 71 currently annotated transmembrane SEPs, only 3 of them (TatE, EcnB and AtpE) were detected in our study. While being identified in the Amphi+ pellet, these 3 proteins showed depletion or remained unidentified in the other Amphi(+) setups. This observation positions the Amphi+ enrichment method as likely suboptimal for global scale investigation of transmembrane SEPs frequently localized in the inner membrane, while intriguingly showing promise in enriching for outer membrane and periplasmic proteins. Nonetheless, in comparison to commonly utilized boiling aided enrichment strategy, the Amphi+ method described here allows for enhanced SEP detection without drastically compromising on the material complexity. Albeit most enrichment procedures disrupt the quantitative aspect of the proteome interrogation, we clearly demonstrate the increased extraction efficiency of SEPs in Amphi+ samples using our optimized protocol, hence their improved MS-based discovery, while nonetheless remaining in good agreement with FPKM expression data at the level of translation (Ribo-seq). In fact, it was observed that, despite effective SEP enrichment the quantitative aspect of the proteomic study was by and large unaffected. This SEP enrichment method might hold the potential to identify a pool of newly discovered and yet uncharacterized SEPs identified in riboproteogenomics efforts (Willems et al., 2020). As demonstrated before (Garai and Blanc-Potard, 2020), small proteins frequently display irregular amino acid compositions and thus different buffer conditions and (MS-) methodologies might be required in order to extract and identify this elusive class of proteins. In our study, significant GO terms enriched in the Amphi+ samples such as “signal peptides” and “outer membrane” suggests the utility of this procedure to also identify secreted and surface displayed small proteins, which are commonly involved in bacterial pathogenesis. Unfortunately, the procedure does not appear suitable for targeting integral inner membrane proteins specifically as a depletion of such proteins could be observed in Amphi+ samples. Since the total amphipol extraction procedure (Amphi) correlates well with classical approaches utilizing high concentrations of chaotropic agents (GuHCl and urea), this drop-in substitute protocol does not have to compromise on the quantitative aspect of proteome study, particularly for larger MW proteins, as long as sufficient material is present and the total proteome can be interrogated alongside the Amphi+ samples using the amphipol based extraction buffer (Amphi). These findings stand in partial opposition with the unbiased snapshot of the proteome obtained using amphipol aided protein precipitation described previously (Ning et al., 2014). Here, we show a clear bias against short proteins and utilize it to specifically enrich for such proteins. With both quantitative and qualitative improvements in the detection of SEPs, this easily adaptable amphipol-based protocol has demonstrated its value for the enrichment of SEPs and thus serves a suitable method for peptidomic screens. In recent years, a great number of alternative proteomic protocols have been proposed to enhance the proteomic detection of SEPs, yet due to a multitude of factors many of them fail in comparison to classical methods coupled with modern, high resolution MS. Ranging from large batch-to-batch variability in filtration material quality observed in size exclusion filtration experiments, through major (and unacceptable) material losses in case of commonly applied boiling strategies, or without an (notable) increase in the number of identified SEPs for some investigated enrichment strategies, simple adaptable protocols enhancing SEP discovery are a valuable commodity (Zougman et al., 2014; Cassidy et al., 2019). With the commonly used methods displaying strong biases, we propose a simple, yet effective protocol that can complement standard investigations of bacterial proteomes, and by extension proteomes of other non-bacterial species.

Small proteins are increasingly in the spotlight of molecular biology, and their identification, validation and functional characterization is central to improving our understanding of bacterial biology. Given the socioeconomic burden of raising antibiotic resistance this research avenue, potentially leading to highlighting future points of therapeutic intervention, remains highly relevant. Recent advancements in genomics strongly suggest that the true complexity of bacterial genomes is still vastly underappreciated (Giess et al., 2017; Ndah et al., 2017; Meydan et al., 2019). With commonly employed proteomic strategies ill-equipped to tackle this challenge, conditional and complementary approaches are needed. In conjunction with more specialized and large-scale efforts recently described, we believe that the proposed and widely applicable protocol may aid to shed light on the elusive nature of SEPs.

## 5 Significance

This study reports on an optimized proteomics protocol facilitating enhanced detection of short ORF encoded proteins (SEPs). With growing evidence of the biological relevance of SEPs and their consistently understudied nature, enrichment protocols like the one proposed in our work allow for validation efforts to progress and shed light on the underlying biology of SEPs. In this study, we exploit the properties of amphipatic polymers, namely amphipol A8-35, allowing for efficient precipitation of larger proteins upon acidification, effectively enriching for small proteins. We compared this approach with several commonly employed protocols used for global proteome interrogation and provide evidence for efficient SEP enrichment. Moreover, we demonstrate improved identification rates and coverage of SEPs using our protocol and point towards potential biases complicating proteomic detection of such proteins. With the mounting evidence supporting biological relevance of small proteins, our easily adoptable protocol can further propel the research investigating this elusive protein class.

## Data Availability

The data presented in the study are deposited in the PRIDE repository, accession number PXD025297.

## References

[B1] Baek J. Lee J. Yoon K. Lee H. (2017). Identification of Unannotated Small Genes in Salmonella. G3 (Bethesda) 7 (3), 983–989. 10.1534/g3.116.036939 28122954 PMC5345727

[B2] Bartel J. Varadarajan A. R. Sura T. Ahrens C. H. Maaß S. Becher D. (2020). Optimized Proteomics Workflow for the Detection of Small Proteins. J. Proteome Res. 19 (10), 4004–4018. 10.1021/acs.jproteome.0c00286 32812434

[B3] Brunet M. A. Brunelle M. Lucier J. F. Delcourt V. Levesque M. Grenier F. (2019). OpenProt: A More Comprehensive Guide to Explore Eukaryotic Coding Potential and Proteomes. Nucleic Acids Res. 47 (D1), D403–D410. 10.1093/nar/gky936 30299502 PMC6323990

[B4] Carr S. Aebersold R. Baldwin M. Burlingame A. Clauser K. Nesvizhskii A. (2004). The Need for Guidelines in Publication of Peptide and Protein Identification Data. Mol. Cell Proteomics 3 (6), 531–533. 10.1074/mcp.t400006-mcp200 15075378

[B5] Cassidy L. Kaulich P. T. Tholey A. (2019). Depletion of High-Molecular-Mass Proteins for the Identification of Small Proteins and Short Open Reading Frame Encoded Peptides in Cellular Proteomes. J. Proteome Res. 18 (4), 1725–1734. 10.1021/acs.jproteome.8b00948 30779583

[B6] Crappé J. Ndah E. Koch A. Steyaert S. Gawron D. De Keulenaer S. (2015). Proteoformer: Deep Proteome Coverage through Ribosome Profiling and MS Integration. Nucleic Acids Res. 43 (5), e29. 10.1093/nar/gku1283 25510491 PMC4357689

[B7] Dinger M. E. Pang K. C. Mercer T. R. Mattick J. S. (2008). Differentiating Protein-Coding and Noncoding RNA: Challenges and Ambiguities. Plos Comput. Biol. 4 (11), e1000176. 10.1371/journal.pcbi.1000176 19043537 PMC2518207

[B8] Fijalkowska D. Fijalkowski I. Willems P. Van Damme P. (2020). Bacterial Riboproteogenomics: The Era of N-Terminal Proteoform Existence Revealed. FEMS Microbiol. Rev. 44 (4), 418–431. 10.1093/femsre/fuaa013 32386204

[B9] Fontaine F. Fuchs R. T. Storz G. (2011). Membrane Localization of Small Proteins in *Escherichia coli* . J. Biol. Chem. 286 (37), 32464–32474. 10.1074/jbc.m111.245696 21778229 PMC3173172

[B10] Fozo E. M. Hemm M. R. Storz G. (2008). Small Toxic Proteins and the Antisense RNAs that Repress Them. Microbiol. Mol. Biol. Rev. 72 (4), 579–589. Table of Contents. 10.1128/mmbr.00025-08 19052321 PMC2593563

[B11] Garai P. Blanc Potard A. (2020). Uncovering Small Membrane Proteins in Pathogenic Bacteria: Regulatory Functions and Therapeutic Potential. Mol. Microbiol. 114 (5), 710–720. 10.1111/mmi.14564 32602138

[B12] Gawron D. Ndah E. Gevaert K. Van Damme P. (2016). Positional Proteomics Reveals Differences in N‐Terminal Proteoform Stability. Mol. Syst. Biol. 12 (2), 858. 10.15252/msb.20156662 26893308 PMC4770386

[B13] Giansanti P. Tsiatsiani L. Low T. Y. Heck A. J. R. (2016). Six Alternative Proteases for Mass Spectrometry-Based Proteomics beyond Trypsin. Nat. Protoc. 11 (5), 993–1006. 10.1038/nprot.2016.057 27123950

[B14] Giess A. Jonckheere V. Ndah E. Chyżyńska K. Van Damme P. Valen E. (2017). Ribosome Signatures Aid Bacterial Translation Initiation Site Identification. BMC Biol. 15 (1), 76. 10.1186/s12915-017-0416-0 28854918 PMC5576327

[B15] Hemm M. R. Paul B. J. Miranda-Ríos J. Zhang A. Soltanzad N. Storz G. (2010). Small Stress Response Proteins in *Escherichia coli* : Proteins Missed by Classical Proteomic Studies. J. Bacteriol. 192 (1), 46–58. 10.1128/jb.00872-09 19734316 PMC2798279

[B16] Hemm M. R. Paul B. J. Schneider T. D. Storz G. Rudd K. E. (2008). Small Membrane Proteins Found by Comparative Genomics and Ribosome Binding Site Models. Mol. Microbiol. 70 (6), 1487–1501. 10.1111/j.1365-2958.2008.06495.x 19121005 PMC2614699

[B17] Hobbs E. C. Yin X. Paul B. J. Astarita J. L. Storz G. (2012). Conserved Small Protein Associates with the Multidrug Efflux Pump AcrB and Differentially Affects Antibiotic Resistance. Proc. Natl. Acad. Sci. 109 (41), 16696–16701. 10.1073/pnas.1210093109 23010927 PMC3478662

[B18] Hu L. Li X. Jiang X. Zhou H. Jiang X. Kong L. (2007). Comprehensive Peptidome Analysis of Mouse Livers by Size Exclusion Chromatography Prefractionation and NanoLC−MS/MS Identification. J. Proteome Res. 6 (2), 801–808. 10.1021/pr060469e 17269736

[B19] Ibrahim M. Nicolas P. Bessières P. Bolotin A. Monnet V. Gardan R. (2007). A Genome-Wide Survey of Short Coding Sequences in Streptococci. Microbiology (Reading) 153 (Pt 11), 3631–3644. 10.1099/mic.0.2007/006205-0 17975071

[B20] Ingolia N. T. Ghaemmaghami S. Newman J. R. S. Weissman J. S. (2009). Genome-Wide Analysis *In Vivo* of Translation with Nucleotide Resolution Using Ribosome Profiling. Science 324 (5924), 218–223. 10.1126/science.1168978 19213877 PMC2746483

[B21] Käll L. Canterbury J. D. Weston J. Noble W. S. MacCoss M. J. (2007). Semi-Supervised Learning for Peptide Identification from Shotgun Proteomics Datasets. Nat. Methods 4 (11), 923–925. 10.1038/nmeth1113 17952086

[B22] Kaulich P. T. Cassidy L. Bartel J. Schmitz R. A. Tholey A. (2021). Multi-protease Approach for the Improved Identification and Molecular Characterization of Small Proteins and Short Open Reading Frame-Encoded Peptides. J. Proteome Res. 20 (5), 2895–2903. 10.1021/acs.jproteome.1c00115 33760615

[B23] Klein C. Aivaliotis M. Olsen J. V. Falb M. Besir H. Scheffer B. (2007). The Low Molecular Weight Proteome of Halobacterium Salinarum. J. Proteome Res. 6 (4), 1510–1518. 10.1021/pr060634q 17326674

[B24] Kyte J. Doolittle R. F. (1982). A Simple Method for Displaying the Hydropathic Character of a Protein. J. Mol. Biol. 157 (1), 105–132. 10.1016/0022-2836(82)90515-0 7108955

[B25] Lluch-Senar M. Delgado J. Chen W. H. Lloréns-Rico V. O'Reilly F. J. Wodke J. A. (2015). Defining a Minimal Cell: Essentiality of Small ORFs and ncRNAs in a Genome-Reduced Bacterium. Mol. Syst. Biol. 11 (1), 780. 10.15252/msb.20145558 25609650 PMC4332154

[B26] Ma J. Diedrich J. K. Jungreis I. Donaldson C. Vaughan J. Kellis M. (2016). Improved Identification and Analysis of Small Open Reading Frame Encoded Polypeptides. Anal. Chem. 88 (7), 3967–3975. 10.1021/acs.analchem.6b00191 27010111 PMC4939623

[B27] Ma J. Ward C. C. Jungreis I. Slavoff S. A. Schwaid A. G. Neveu J. (2014). Discovery of Human sORF-Encoded Polypeptides (SEPs) in Cell Lines and Tissue. J. Proteome Res. 13 (3), 1757–1765. 10.1021/pr401280w 24490786 PMC3993966

[B28] Meydan S. Marks J. Klepacki D. Sharma V. Baranov P. V. Firth A. E. (2019). Retapamulin-Assisted Ribosome Profiling Reveals the Alternative Bacterial Proteome. Mol. Cel 74 (3), 481–493. 10.1016/j.molcel.2019.02.017 PMC7115971 30904393

[B29] Miravet-Verde S. Ferrar T. Espadas-García G. Mazzolini R. Gharrab A. Sabido E. (2019). Unraveling the Hidden Universe of Small Proteins in Bacterial Genomes. Mol. Syst. Biol. 15 (2), e8290. 10.15252/msb.20188290 30796087 PMC6385055

[B30] Monera O. D. Kay C. M. Hodges R. S. (1994). Protein Denaturation with Guanidine Hydrochloride or Urea Provides a Different Estimate of Stability Depending on the Contributions of Electrostatic Interactions. Protein Sci. 3 (11), 1984–1991. 10.1002/pro.5560031110 7703845 PMC2142645

[B31] Ndah E. Jonckheere V. Giess A. Valen E. Menschaert G. Van Damme P. (2017). Reparation: Ribosome Profiling Assisted (Re-)Annotation of Bacterial Genomes. Nucleic Acids Res. 45 (20), e168. 10.1093/nar/gkx758 28977509 PMC5714196

[B32] Ning Z. Hawley B. Seebun D. Figeys D. (2014). APols-Aided Protein Precipitation: A Rapid Method for Concentrating Proteins for Proteomic Analysis. J. Membr. Biol. 247 (9-10), 941–947. 10.1007/s00232-014-9668-6 24838764 PMC4196042

[B33] Olexiouk V. Crappé J. Verbruggen S. Verhegen K. Martens L. Menschaert G. (2016). sORFs.org: A Repository of Small ORFs Identified by Ribosome Profiling. Nucleic Acids Res. 44 (D1), D324–D329. 10.1093/nar/gkv1175 26527729 PMC4702841

[B34] Omasits U. Varadarajan A. R. Schmid M. Goetze S. Melidis D. Bourqui M. (2017). An Integrative Strategy to Identify the Entire Protein Coding Potential of Prokaryotic Genomes by Proteogenomics. Genome Res. 27 (12), 2083–2095. 10.1101/gr.218255.116 29141959 PMC5741054

[B35] Petruschke H. Anders J. Stadler P. F. Jehmlich N. von Bergen M. (2020). Enrichment and Identification of Small Proteins in a Simplified Human Gut Microbiome. J. Proteomics 213, 103604. 10.1016/j.jprot.2019.103604 31841667

[B36] Popot J.-L. Althoff T. Bagnard D. Banères J.-L. Bazzacco P. Billon-Denis E. (2011). Amphipols from A to Z*. Annu. Rev. Biophys. 40, 379–408. 10.1146/annurev-biophys-042910-155219 21545287

[B37] Popot J.-L. (2010). Amphipols, Nanodiscs, and Fluorinated Surfactants: Three Nonconventional Approaches to Studying Membrane Proteins in Aqueous Solutions. Annu. Rev. Biochem. 79, 737–775. 10.1146/annurev.biochem.052208.114057 20307193

[B38] Rowland S. L. Burkholder W. F. Cunningham K. A. Maciejewski M. W. Grossman A. D. King G. F. (2004). Structure and Mechanism of Action of Sda, an Inhibitor of the Histidine Kinases that Regulate Initiation of Sporulation in Bacillus Subtilis. Mol. Cel 13 (5), 689–701. 10.1016/s1097-2765(04)00084-x 15023339

[B39] Saghatelian A. Couso J. P. (2015). Discovery and Characterization of smORF-Encoded Bioactive Polypeptides. Nat. Chem. Biol. 11 (12), 909–916. 10.1038/nchembio.1964 26575237 PMC4956473

[B40] Samayoa J. Yildiz F. H. Karplus K. (2011). Identification of Prokaryotic Small Proteins Using a Comparative Genomic Approach. Bioinformatics 27 (13), 1765–1771. 10.1093/bioinformatics/btr275 21551138 PMC3117347

[B41] Shaw K. L. Grimsley G. R. Yakovlev G. I. Makarov A. A. Pace C. N. (2001). The Effect of Net Charge on the Solubility, Activity, and Stability of Ribonuclease Sa. Protein Sci. 10 (6), 1206–1215. 10.1110/ps.440101 11369859 PMC2374010

[B42] Staes A. Van Damme P. Helsens K. Demol H. Vandekerckhove J. Gevaert K. (2008). Improved Recovery of Proteome-Informative, Protein N-Terminal Peptides by Combined Fractional diagonal Chromatography (COFRADIC). Proteomics 8 (7), 1362–1370. 10.1002/pmic.200700950 18318009

[B43] Tyanova S. Temu T. Cox J. (2016). The MaxQuant Computational Platform for Mass Spectrometry-Based Shotgun Proteomics. Nat. Protoc. 11 (12), 2301–2319. 10.1038/nprot.2016.136 27809316

[B44] Tyanova S. Temu T. Sinitcyn P. Carlson A. Hein M. Y. Geiger T. (2016). The Perseus Computational Platform for Comprehensive Analysis of (Prote)omics Data. Nat. Methods 13 (9), 731–740. 10.1038/nmeth.3901 27348712

[B45] Venturini E. Svensson S. L. Maaß S. Gelhausen R. Eggenhofer F. Li L. (2020). A Global Data-Driven Census of Salmonella Small Proteins and Their Potential Functions in Bacterial Virulence. microLife 1 (1), uqaa002. 10.1093/femsml/uqaa002 PMC10117436 37223003

[B46] Wadler C. S. Vanderpool C. K. (2007). A Dual Function for a Bacterial Small RNA: SgrS Performs Base Pairing-Dependent Regulation and Encodes a Functional Polypeptide. Proc. Natl. Acad. Sci. 104 (51), 20454–20459. 10.1073/pnas.0708102104 18042713 PMC2154452

[B47] Warren A. S. Archuleta J. Feng W.-C. Setubal J. C. (2010). Missing Genes in the Annotation of Prokaryotic Genomes. BMC Bioinformatics 11, 131. 10.1186/1471-2105-11-131 20230630 PMC3098052

[B48] Willems P. Fijalkowski I. Van Damme P. (2020). Lost and Found: Re-Searching and Re-Scoring Proteomics Data Aids Genome Annotation and Improves Proteome Coverage. mSystems 5 (5), e00833–20. 10.1128/mSystems.00833-20 33109751 PMC7593589

[B49] Zhu Y. Orre L. M. Johansson H. J. Huss M. Boekel J. Vesterlund M. (2018). Discovery of Coding Regions in the Human Genome by Integrated Proteogenomics Analysis Workflow. Nat. Commun. 9 (1), 903. 10.1038/s41467-018-03311-y 29500430 PMC5834625

[B50] Zougman A. Selby P. J. Banks R. E. (2014). Suspension Trapping (STrap) Sample Preparation Method for Bottom-Up Proteomics Analysis. Proteomics 14 (9), 1006–1000. 10.1002/pmic.201300553 24678027

