# Supplementary figures and images for: Small Protein Enrichment Improves Proteomics Detection of sORF Encoded Polypeptides
Source: Front Genet. 2021 Oct 15;12:713400. doi: 10.3389/fgene.2021.713400 (PMC8554064; doi:10.3389/fgene.2021.713400)

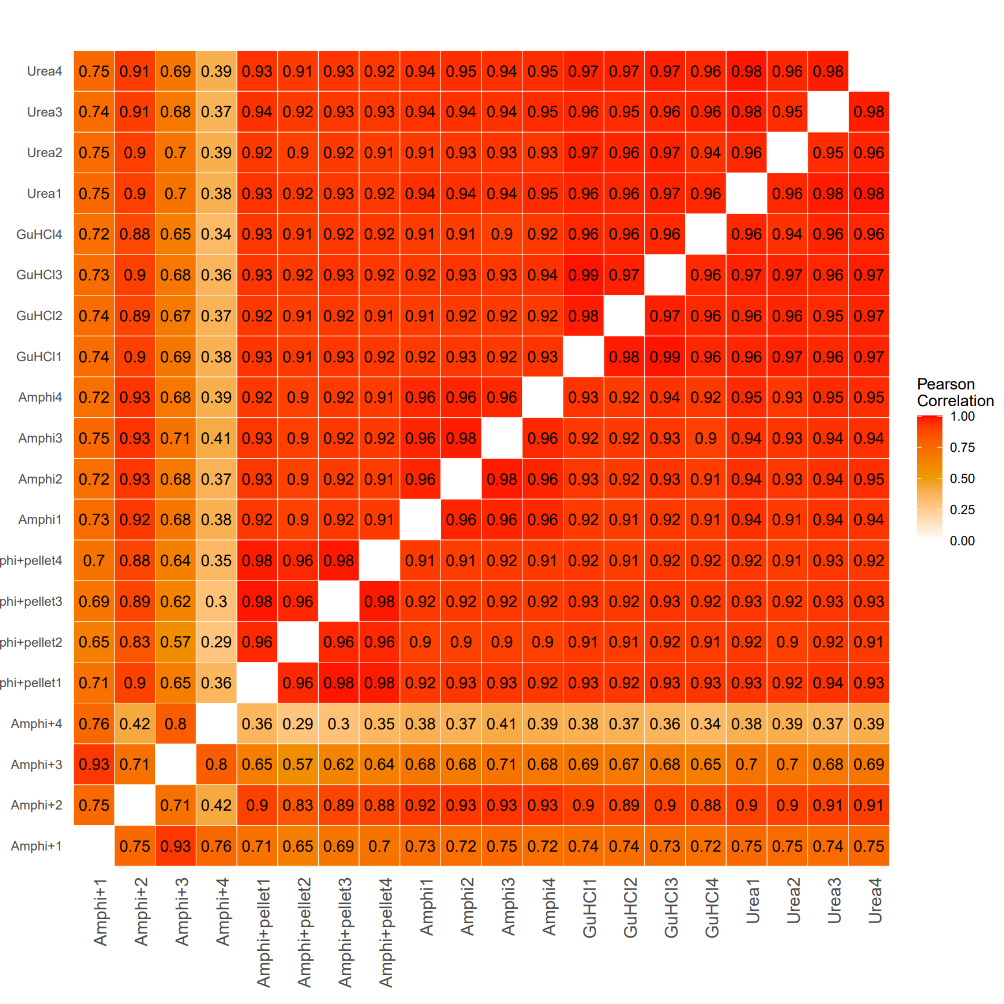

Supplement: Supplementary file 2 [file Image1.TIFF]
